# Innovative strategies in bile duct repair: Assessing efficacy and safety across varied graft techniques - A systematic review

**DOI:** 10.1016/j.sopen.2025.01.006

**Published:** 2025-01-25

**Authors:** Anung Noto Nugroho, Soetrisno Soetrisno, Ambar Mudigdo, Kristanto Yuli Yarso, Dono Indarto, Akmal Zhahir Wahyudi, Enrico Ananda Budiono, Auliya Yudia Yasyfin

**Affiliations:** a Doctoral Program of Medical Sciences, Faculty of Medicine, Sebelas Maret University, Surakarta 57126, Jawa Tengah, Indonesia; b Obstetrics and Gynecology Department, Dr. Moewardi Hospital/Faculty of Medicine, Sebelas Maret University, Surakarta 57161, Jawa Tengah, Indonesia; c Department of Anatomical Pathology, Dr. Moewardi Hospital/Faculty of Medicine, Sebelas Maret University, Surakarta 57126, Jawa Tengah, Indonesia; d Oncology Division, Surgery Department, Sebelas Maret University, Surakarta 57126, Jawa Tengah, Indonesia; e Department of Physiology and Biomedical Laboratory, Sebelas Maret University, Surakarta, Jawa Tengah, Indonesia; f Faculty of Medicine, Sebelas Maret University, Surakarta 57126, Jawa Tengah, Indonesia

**Keywords:** Bile duct injury, Graft, Surgical procedure, Animal model, Postoperative complication

## Abstract

Bile duct injuries (BDI) from surgical procedures pose significant clinical challenges, requiring precise interventions for optimal outcomes. This systematic review explores the utilization of grafts in the repair of bile duct injuries, aiming to gain insights from existing literature. Graft-based techniques show promise in improving postoperative outcomes, but their efficacy varies. A systematic search was conducted across PubMed, Science Direct, and Scopus following the PRISMA 2020 Checklist, focusing on studies published until February 19, 2024. The inclusion criteria involved research using grafts to treat bile duct injuries in pig, swine, or mini-pig models. Out of 2231 studies identified, eleven met the inclusion criteria. These studies evaluated various graft techniques, including autologous tissue with biodegradable stents, decellularized grafts, patches, prosthetic grafts, bacterial cellulose film, and heterogeneous materials. Each method had distinct advantages and limitations, particularly regarding postoperative outcomes and histological findings. This review highlights the need for further research to determine the most effective graft-based strategies for BDI repair and improve patient care.

## Introduction

Bile duct injuries (BDI) resulting from surgical procedures represent a significant burden in clinical practice, with the incidence of post-cholecystectomy BDI ranging from 0.1 % to 1.5 % in recent literature, and a median incidence of 0.5 %. Notably, this rate has shown no decrease in recent years, indicating an ongoing clinical challenge. In France alone, approximately 600 patients are affected by post-cholecystectomy BDI each year [1]. In the United States, bile duct injury occurs in 0.3 % to 0.7 % of the roughly 750,000 laparoscopic cholecystectomies performed annually. These injuries are a significant cause of morbidity and mortality, particularly when they are not recognized intraoperatively—a situation that occurs only 25 % to 32.4 % of the time [2]. The clinical landscape is further complicated by the diversity of injury types, ranging from minor leaks to complete transections, each necessitating tailored approaches to repair [3]. Despite advancements in surgical techniques, BDI remains a challenge due to its potential for severe complications, including bile peritonitis, strictures, cholangitis, and liver failure. These complications affect patient outcomes and contribute to increased healthcare costs and prolonged hospital stays [4]. Addressing the challenges of bile duct injuries requires innovative strategies that ensure efficacy and safety while reducing complications and improving long-term patient outcomes. Existing research on bile duct repair techniques has predominantly focused on traditional methods, leaving a gap in understanding the potential of emerging graft-based approaches. Furthermore, the limited translation of promising findings from animal models to clinical practice underscores the need for systematic evaluation. This review critically assesses various graft techniques used in bile duct injury repair, synthesizing evidence to inform clinical practice and guide future research endeavors.

## Materials and methods

### Search strategy

This study presents a systematic review following the PRISMA 2020 Checklist. The search was conducted on February 19, 2024, across three global databases - PubMed, Science Direct, and Scopus - aligned with the study's objectives. We formulated the PICO question based on specific criteria: population - including pigs, swine, or mini pigs with bile duct injuries in both genders; intervention - encompassing the use of various grafts as the primary approach for treating bile duct injuries, including stents, patches, and artificial ducts; comparison - comparing bile duct injury repair with and without grafts (e.g., primary closures); outcome - evaluating both effectiveness and safety through parameters such as complete blood count, liver enzymes, and histopathological analysis. Keywords used included (“bile duct injury” OR “bile duct injuries” OR “biliary tract injury” OR “biliary tract injuries”) AND (graft OR autologous OR autograft OR autografting OR “autologous transplantation” OR “autologous transplant”). To ensure comprehensive coverage, we conducted a manual search of the references cited in the included studies following a systematic search to identify relevant literature.

### Inclusion and exclusion criteria

The subsequent step involved establishing criteria for inclusion and exclusion. Inclusion criteria included studies involving the use of various grafts as the primary method for treating bile duct injuries in pig, swine, or mini-pig models of both sexes. Included studies had to involve a minimum of five subjects and be published in English regardless of publication year, with no restrictions on country or follow-up duration. Exclusion criteria included research employing human models or animals with pregnancies or comorbidities, those using heterologous grafts for injury therapy, in vitro studies, review papers, case reports, case series, and abstracts.

### Selection process

In the selection process, titles and abstracts of potential applicable research were assessed independently by three reviewers (AZW, AYY, and EAB). The inclusion and exclusion criteria were applied to the identification and assessment of full-text papers for eligibility. If there were any conflicts among the reviewers, they were resolved by a process of mutual discussion, followed by the agreement of the fourth reviewer (ANN).

### Quality assessment

SYRCLE's risk of bias tool was used to evaluate the quality of the qualifying articles. This tool has ten primary domains: sequence generation, baseline characteristics, allocation concealment, random housing, investigator blinding, random outcome assessment, blinding of outcome, incomplete outcome data, selective outcome reporting, and other sources of bias. The outcomes of the bias assessment were presented through traffic-light plot and summary plot graphs, depicting the levels of low, high, or unclear risk of bias [5].

### Data analysis

The data analysis in this systematic review involved summarizing the findings of the included studies using a narrative synthesis approach. The obtained data was subjected to descriptive analysis, with continuous outcomes represented by the mean and standard deviation (SD) and categorical outcomes defined by frequencies and percentages. The selected papers were evaluated qualitatively for similar patterns and themes in their intervention techniques as well as the outcomes of the studies. Publication bias was not evaluated because this study did not do a meta-analysis.

## Results

### Study selections

**Fig. 1:**
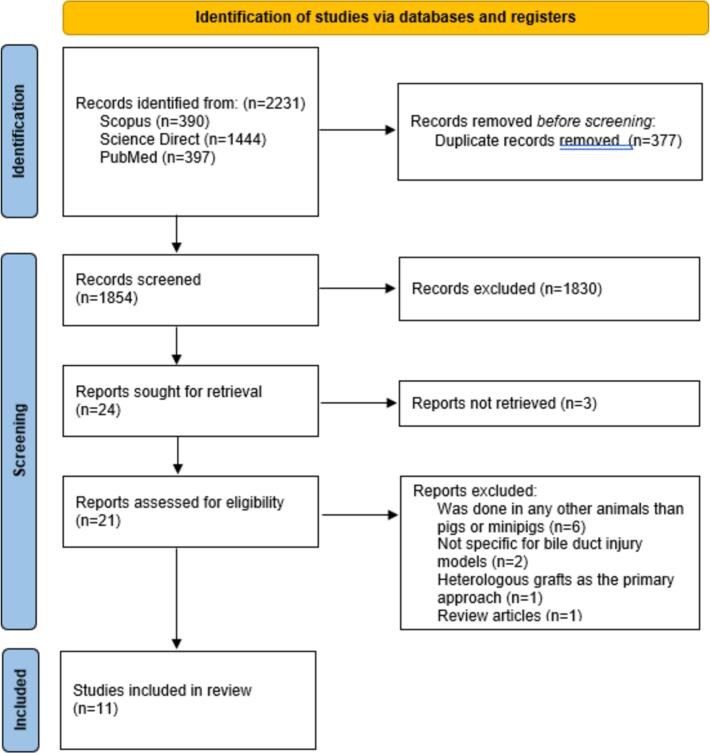
Flow diagram of the study based on PRISMA 2020 guideline [6].

The PRISMA diagram that accompanies Fig. 1 provides an overview of the gathering technique. A total of 2231 studies were obtained from the following databases: Scopus: 390 records, Science Direct: 1444 records, and PubMed: 397 entries. Before the screening was completed, 377 duplicate records were eliminated. Following the screening of the title and abstract, 1830 papers were excluded from the research. Following the inability to get three complete texts, the entire texts of twenty-one publications were reviewed. Six research papers were conducted in animals other than pigs or minipigs; two studies were not specifically designed for bile duct damage models; one study employed heterologous grafts as the major strategy; and one was a review article. As the final studies for the qualitative synthesis, the final eleven articles were selected.

### Quality and risk of bias

**Fig. 2:**
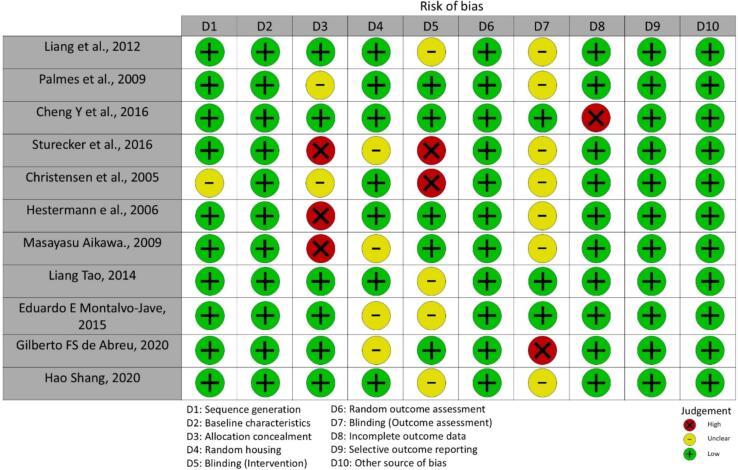
Graph summary of the risk of bias assessment tool for animal studies.

SYRCLE's risk of bias tool was utilized to assess the papers selected for a more thorough analysis. The instrument has been used in research involving animal experiments. The following ten domains were evaluated based on the response: yes, no, and unclear. The answers “yes” denote a low risk of bias, “no” a high risk of bias, and “unclear” an unclear risk of bias. These characteristics will be evaluated and interpreted independently [5]. Fig. 2 depicts the risk of skewed results using a traffic-light plot.

### Study characteristics

There were a total of 150 samples from the eleven experimental studies that were included, with sample sizes ranging from 5 to 24 animals. The longest follow-up times range from eight days to six months, after which the individuals are killed off to examine the results in full. The animals employed range in weight from 15 to 60 kg and include Ba-Ma mini-pigs, German Landrace pigs, Neijiang Cross porcine, Danish Landrace pigs, Deutsche Landrasse pigs, Piau Suidae swine, and domestic hybrid pigs. Based on the type of pigs utilized, resection from 0.5–4 cm to the duct is done to create models of bile duct injuries. The reviewed papers utilized repair techniques utilizing autologous tissue combined with biodegradable stents, decellularized grafts, patch, prosthetic grafts, bacterial film, and heterogeneous materials. There are several different approaches to bile duct restoration, but most involve the use of autologous tissue, stents, patches, and bacterial cellulose film. Table 1 displays the whole characteristic description for the studies. Eleven studies were included in the analysis; three of them combined the use of autologous tissue with biodegradable stents; two of them used decellularized grafts; two of them utilized patches; two of them used prosthetic grafts; one study used a bacterial film; and the other study used heterogeneous material.

**Table 1:** Characteristic description of the included studies.

No	Author (year of study)	Aim of study	Samples (species, no. of species, groups)	No. of samples	Injury model	Study duration	Intervention	Results and conclusion
1	Liang et al. (2012) [7]	To propose and assess a novel technique for repairing bile duct defects using a degradable stent combined with autologous tissues	*Ba-Ma* mini-pigs Either sex Weighing 15–20 kg	8	0.5–1.0 cm segment of CBD resected.	1 year follow-up Postoperative evaluations in the 1st and 3rd months	The defect was repaired through duct-to-duct anastomosis using a	All animals survived surgery without bile leakage or mortality. The stent maintained shape and position at 1 month, disappearing by 3 months, with no bile duct issues observed. No complications like necrosis or stricture occurred, though one pig had intestinal obstruction at 4 months. The novel bile duct repair method showed promising short-term outcomes, but long-term effects require further study.
2	Palmes et al. (2009) [8]	To examine the morphological characteristics of a neo-bile duct formed using a vein and a biodegradable endoluminal stent.	*German Landrace* Female pigs Weighing 20–25 kg Divided equally into four groups of intervention	24	A 2 cm long segment of CBD was resected	6 months follow-up	A segment of the CBD was replaced with a segment of the external jugular vein, which was either supported by a biodegradable poly-lactate-acid (PLA) stent or left unsupported	Over six months, the neo-bile duct developed to resemble the native bile duct, with Ck7-positive columnar epithelium and new capillaries. The biodegradable stent dissolved by four months. All animals survived with normal liver function and no cholestasis. However, vein reconstruction alone led to four deaths from biliary peritonitis and cholangitis.
3	Cheng Y et al. (2016) [9]	To assess the practicality of using a decellularized ureteral graft to repair a common bile duct defect in a porcine model.	Porcine model (*Neijiang cross*) Either sex Weighing 25–35 kg Divided equally into three groups of intervention	18	A 1 cm long segment distal to the entrance of the cystic duct was resected	3 months follow-up	The intervention involved the repair of a common bile duct defect using a 2 cm long decellularized ureteral graft with either a T-tube, a silicone stent, or no stent (stentless group)	Animals in the T-tube and stent groups survived with normal blood tests and a biliary epithelial layer forming over the neo-bile duct. In contrast, all stentless group animals died from biliary peritonitis and cholangitis within two months, with no epithelial cells or glands at the graft sites. Repairing a CBD defect with a decellularized ureteral graft is feasible, but a T-tube or intraluminal stent is necessary to prevent complications.
4	Struecker et al. (2016) [10]	To assess the potential of an allogeneic aorta into a syngeneic bile duct in vitro and subsequently implant it orthotopically in vivo.	Domestic pigs Either sex Weighing 40–60 kg	5	3–4 cm of the native extrahepatic bile duct was resected	14 days follow-up	A bioengineered neo-bile duct, created through decellularization and recellularization, was implanted in domestic pigs to replace the native bile duct	All pigs survived the study without severe complications, showing no biliary leakage or peritonitis. The neo-bile ducts had neutrophil infiltration and neoangiogenesis in the implanted tissue. This study introduces a new approach for extrahepatic bile duct replacement using an ex vivo-generated autologous neo-bile duct, but its long-term effectiveness needs further evaluation.
5	Christensen et al. (2005) [11]	To explore the feasibility of using a vascular graft for reconstructing the CBD in pigs.	*Danish Landrace*/*Yorkshire* Female pigs Weighing 60 kg	8	The cystic duct and the cystic artery were ligated and divided (length not stated).	8 days follow-up	The CBD was reconstructed using a standard expanded polytetrafluoroethylene (ePTFE) vascular graft	By the 8th postoperative day, all but one pig showed no bile leakage; one had a biloma, and another underwent surgery on day 6 for a gastric ulcer without bile leakage. Cholangiography revealed slight intrahepatic dilation, but bilirubin and alkaline phosphatase levels were stable, indicating the short-term safety of vascular graft reconstruction in pigs.
6	Heistermann et al. (2006) [12]	Detail a novel method for bile duct reconstruction in a pig model using an autologous vein graft supported by an endoluminal biodegradable polylactate acid stent.	*Deutsche Landrasse* Female pigs Weighing 20–25 kg	12	A 2 cm-long segment of the CBD was resected	3–6 months follow-up	The defect was repaired using an autologous vein graft supported by a biodegradable endoluminal stent	All pigs in the survival group lived for 6 months before being sacrificed. By 4 months, the stent had fully degraded, and the vein graft was relined with bile duct epithelium. This technique for bile duct reconstruction using an autologous vein graft with an endoluminal stent is simple, reliable, and offers a promising alternative to bilo-digestive anastomosis by preserving the papilla of Vateri.
7	Masayasu Aikawa (2009) [13]	To explore the potential of a bioabsorbable polymer (BAP) patch as a new treatment option for biliary stenosis.	Hybrid pigs Weighing 15–30 kg 1–2 years age	12	A spindle-shaped excision was done measuring 2 × 0.6 cm of the lower CBD wall	4 months follow-up Postoperative evaluations in the 5th week and 4th months	A bile duct defect was repaired using a tissue-engineered bioabsorbable polymer patch, designed to degrade and facilitate tissue regeneration	All recipient pigs survived until the end, gained weight, and showed no signs of jaundice. The BAP-patched duct remained unobstructed at 5 weeks, with normal hepatobiliary enzyme levels. Histology revealed glandular structures in the neo-bile duct, and by 4 months, the graft site was indistinguishable from the native duct. Intra-operative cholangiography showed localized dilation at the patch site, but no intrahepatic bile duct dilation. The bile duct had focal dilation only at the implantation site. This newly designed substitute shows promise as a novel treatment for biliary injury and stenosis.
8	Liang Tao (2015) [14]	To assess the effectiveness of a collagen membrane used as a patch for reconstructing a spindle-shaped defect on the extrahepatic bile duct (EBD) in a swine model.	Hybrid pigs of both sexes Weighing 15–20 kg	20	A spindle-shaped excision was done measuring 2 × 0.6 cm of the lower CBD wall	3 months follow-up Postoperative evaluations on the 2nd, 4th, 8th, and 12th weeks	A collagen patch was used to repair a spindle-shaped defect in the extrahepatic bile duct of pigs, supported by a drainage tube and wrapped with greater omentum	No leakage or stricture was observed, though some pigs developed biliary sludge or stones at 4 and 8 weeks. The drainage tube was lost within 12 weeks. The neo-EBD withstood normal biliary pressure 2 weeks post-surgery. Histology showed gradual regeneration of accessory glands and epithelial cells, increased vessel infiltration, and reduced inflammation. Collagen fibers became regular with complete epithelial coverage. Statistical analysis showed no stricture at the graft site, though the EBD wall was slightly thicker due to collagen deposition. The neo-EBD structure resembled the normal EBD. The collagen membrane patch with a drainage tube and greater omentum effectively promoted EBD defect regeneration within 12 weeks.
9	Eduardo E Montalvo-Jave (2015) [15]	To showcase the effectiveness of a polymer-based absorbable bioprosthesis incorporating a bone collagen scaffold for treating bile duct injuries in an animal model.	Pigs, Landrace pigs Male pigs Weighing 30–40 kg Divided equally into three groups	15	A transverse section (length not stated) of the CBD with a proximal and distal anastomosis (CBD-bioprosthesis-CBD) was done with a simple suture.	6 months follow-up Postoperative evaluations at 1st, 3rd, and 6th months	An absorbable bioprosthesis with a bone collagen scaffold was used to replace the common bile duct in pigs, aiming to support tissue regeneration without postoperative complications.	Liver function tests showed no significant changes at various time points, and no mortality or postoperative complications occurred in any models. Imaging studies (MRCP, ERCP, and Choledochoscopy with SpyGlass™) revealed no stenosis or obstruction. Histology and immunohistochemistry (CK19 and MUC5+) confirmed the presence of biliary epithelium with intramural biliary glands in all models. The bioprosthesis provided effective scaffolding for tissue regeneration, with no complications at 6 months follow-up. This bioprosthesis is suitable for replacing bile ducts in cases of cancer or injury and can be adapted for different types of bile duct damage.
10	Gilberto FS de Abreu (2020) [16]	To assess the effectiveness of bacterial cellulose film and bile duct autograft in repairing severe common bile duct injuries in pigs.	*Sus domesticus*, *Piau suidae* swine Divided into two groups	20	A median abdominal incision was done in the CBD. In the CG, two complete critical CBD sections are 10 mm apart. In the EG, a 10 mm section of the longitudinal shaft with edge resection.	330 days follow-up Postoperative evaluations at 150th, 225th, and 330th days	A bacterial cellulose film was used to repair a critical common bile duct injury in pigs, demonstrating biocompatibility and complete healing without bile duct dilation or significant liver dysfunction.	Intraoperative ultrasonography revealed significant ductal narrowing and obstruction in the control group after common bile duct anastomosis. The bacterial cellulose film E2 group showed increased inflammation, granulomatous reaction, fibrosis, and vessel density, but no bile duct dilation on ultrasound. Biochemical analysis of liver enzymes remained normal, indicating preserved liver function at various post-surgery time points. Bacterial cellulose film proved to be a biocompatible graft material, facilitating complete healing and maintaining biliary flow continuity.
11	Hao Shang (2020) [17]	To assess the long-term viability of a new heterogeneous ada-BD for treating extrahepatic bile duct injuries in pigs.	Guangxi Bama minipigs Either sex Weighing 25–35 kg Divided into two groups	8	A 2-cm long segment of CBD was resected	6–12 months follow-up	A novel animal-derived artificial bile duct (ada-BD) was used to repair a 2 cm segmental defect in the common bile duct of pigs, facilitating bile duct regeneration without immune rejection	The median operative time was 2.45 h, with a 60.5-minute anastomosis. All animals survived, with stable liver function. Histology showed a well-formed biliary epithelial layer and regeneration of connective tissue and smooth muscle, without significant immune rejection. Group B had thicker, more regular connective tissue and better epithelial regeneration, though Group A showed superior smooth muscle regeneration. The ada-BD graft approach appears feasible for CBD repair, but larger studies are needed to confirm its long-term effectiveness.

## Discussion

This systematic review evaluates 11 studies on graft techniques for Bile Duct Injury (BDI) using pigs as animal models. Pigs are widely utilized due to their anatomical and physiological similarities to humans, particularly in biliary tract structure, immune response, and healing processes, which make them suitable for studying surgical techniques. Their size accommodates standard surgical instruments, enabling detailed experimentation and postoperative evaluation. However, the high maintenance costs and anatomical differences, such as variations in bile duct structure, may limit direct applicability to humans. Despite these drawbacks, pigs remain a reliable model for advancing bile duct repair research.

While the use of animal models, particularly pigs, has provided valuable insights into bile duct repair techniques due to their anatomical and physiological similarities to humans, the transition to clinical applications requires careful consideration. Future research should focus on validating these techniques in preclinical humanized models and conducting clinical trials to assess safety, efficacy, and long-term outcomes in human patients. Key areas for translation include addressing challenges related to immune responses, optimizing graft materials for human use, and ensuring scalability of production for clinical settings. Additionally, regulatory pathways must be navigated to ensure that these novel techniques meet safety and efficacy standards for human application.

Efforts to find effective alternatives for repairing long-standing bile duct abnormalities caused by illness or trauma have evolved over time. Early studies explored autografts and synthetic grafts as potential solutions for treating common bile duct lesions in situ. Significant advancements were achieved in the 21st century, with the development of degradable materials such as polypropylene, collagen sponge, polyglycolic acid and trimethylene carbonate, poly-lactic acid and polycaprolactone, and small intestinal submucosa [[18], [19], [20], [21], [22]]. Despite these innovations, until recently, no replacements had proven reliable and clinically viable for restoring bile duct function.

In this review, the SYRCLE risk of bias tool highlighted notable variability in methodological rigor across the included studies. While most studies demonstrated low risk in domains like sequence generation, baseline characteristics, and random outcome assessment, there were significant gaps in allocation concealment, blinding of interventions, and selective outcome reporting. These weaknesses, particularly in blinding and allocation methods, may have introduced bias that could affect the reliability of the reported outcomes. The lack of standardized methodological practices underscores the need for greater consistency and transparency in preclinical research to enhance the quality and applicability of findings.

### Autologous tissue combined with biodegradable stents

One of the promising novel techniques for bile duct injury was the development of degradable stents. These stents were used to cover the disadvantages shown by the prior study which used an autologous vein segment for bile duct injury which leads to the development of the bile duct strictures, necrosis, or stenosis [23]. However, other research also showed that the usage of plastic or metal stents showed considerable problems, such as bile duct lesions by stent insertion, foreign body reaction, fistula formation, stent occlusion, or migration [[23], [24], [25], [26]].

The effectiveness of combining autologous tissue with biodegradable stents was further explored. These stents dissolve on their own, eliminating the need for removal surgery and reducing complications like migration or kinking [23,27]. Liang et al. (2012) used a degradable inner stent made of PSPP to prevent restenosis, which degrades within 2 to 3 months [7,28]. Large omentum tissue was also used to enhance blood supply. The plasma concentration of γ-­GT showed a significant rise following the surgical procedure particularly two weeks post-operation, which suggests the occurrence of liver injury. Pathological examination showed no significant cholestasis present in the liver tissue, as collagen deposition and proliferation of bile duct glands were detected in the anastomosis using hematoxylin and eosin (HE) as well as Masson's trichrome stains. This research's combination resulted in stable bilirubin and ALT levels after 12 months, with no bile duct issues (dilatation, stenosis, bile leakage, or any sign of biliary stricture) observed.

Another study by Palmes et al. (2009) paired an autologous vein with a biodegradable PLA stent, acting as a temporary scaffold [8]. The study showed that combinations of the materials were superior compared to the usage of autologous veins alone. AST, bilirubin, AP, albumin, leukocytes, and γ-­GT were shown to be within the normal range in the newly formed bile duct. Within four months post-operation, the neo-bile duct was covered with bile duct epithelium and the stent had fully degraded. Two months later, the neo-bile duct closely resembled the native one, displaying Ck-7 positive columnar epithelium and newly formed capillaries in the duct wall. Conversely, using only the vein led to necrosis, biliary peritonitis, or bile duct stenosis. The degradable stent prevented vein collapse and protected it from alkaline bile, facilitating capillary and bile duct epithelium growth. However, stent size is critical; too large a stent can cause stenosis, while insufficient length risks anastomosis insufficiency, endangering vein remodeling [8].

Another similar study by Heistermann et al. (2005) found that a vein graft combined with a biodegradable stent showed a histological examination using HE staining revealed that after 3 months, the vein graft was already fully covered with bile duct mucosa. However, there were still remnants of the stent present, which caused an inflammatory reaction and fibrosis. The histological study revealed the anatomy of the normal bile duct after a 6-month observation period [12].

### Decellularized grafts

Another promising approach for treating biliary duct injuries involves using decellularized grafts. This method removes cells and tissue-specific material, allowing for the selective transformation of one tissue type into another by recellularization of the decellularized tissue with a chosen cell type. During this process, all antigenic material must be thoroughly eliminated to prevent harmful host reactions after implantation into different organisms [29]. Recently, various decellularization techniques have been introduced to remove cellular antigens and preserve the extracellular matrix (ECM) [30]. The ECM comprises structural proteins (e.g., collagen, elastin), glycosaminoglycans, and growth factors. Derived from human or animal tissue, ECM biomaterial is biodegradable, decellularized, and non-immunogenic, with proven regenerative and tissue-healing properties [22,30]. Widely used in tissue engineering, ECM biomaterial not only prevents immune rejection and inflammation but also promotes cell adhesion and differentiation for tissue repair and scar prevention [22,30]. Unfortunately, there are currently no defined standards for defining the word “decellularized” that have impacts on experimentation or clinical practice [13]. Consequently, it is still unknown precisely when a tissue or organ is “decellularized” and stops producing harmful host reactions.

In a prior study by Rosen et al. [22], an ECM biomaterial derived from small intestinal submucosa was used to repair bile duct defects without T-tubes or intraluminal stents. However, one out of five dogs developed a biliary stricture two months post-surgery [31]. In a subsequent study, Cheng et al. chose a ureter as the graft due to its thicker smooth muscle layer compared to the small intestine [9]. They used decellularization to create ECM biomaterial. Comparing grafts with and without T-tubes or intraluminal stents, they found that the stentless group experienced severe complications, including graft collapse and biliary stricture formation, as well as a substantial rise in total bilirubin, ALT, and GGT. Cheng suggested that the soft texture of the decellularized ureteral graft couldn't withstand the low pressure in the biliary system, necessitating the use of either a T-tube or intraluminal stent to improve survival rates. However, the use of T-tubes and biliary stents remains controversial, as they may decrease biliary stricture incidence but increase morbidity and complications such as premature dislodgement, biliary leakage, infection, and reduced quality of life [32]. This study also compared a silicone stent with T-tube drainage, which concludes that T-tube drainage was superior to the silicone stent during the bile duct reconstruction in this experiment because the stent was not biodegradable and an additional procedure (e.g., endoscopy, laparotomy) was required to remove the stent.

Struecker et al. (2016) conducted a study using tissue-engineered neo-bile ducts created by decellularizing allogeneic blood vessels and recellularizing them with autologous cholangiocytes [10]. They observed the complete removal of cells from decellularized blood vessels, along with a significant decrease in DNA content over 7 days. Despite variations in DNA content in native vessels, likely due to pig age and size differences and sampling discrepancies, neutrophil infiltration did not result in the expected DNA content increase. This could be attributed to measurement methods and tissue swelling. Neutrophil infiltration suggested possible infection or slowed matrix degradation post-decellularization. Incomplete decellularization might lead to tissue inflammation and abscesses post-implantation [33]. Tissue de- and recellularization could yield non-immunogenic or autologous tissue, possibly negating the need for immunosuppression, though its effect on host response remains uncertain without immunosuppressive administration in recipient pigs. The tissue-engineered Neo-Bile duct showed normal levels of creatinine and ALT. Throughout the trial, all five pigs survived without experiencing any adverse events or complications until the study's conclusion on day 14, except for one pig that had noticeably increased bilirubin.

### Patch

The research conducted by Aikawa et al. (2009) explored the efficacy of a bioabsorbable polymer (BAP) patch in repairing narrowed bile ducts [13]. Unlike previous studies utilizing BAP tubes for bypass grafts, this study focused on the patch's potential for duct repair. The BAP patch, composed of polylactic acid and polycaprolactone, degraded within 6–8 weeks, facilitating cell penetration due to its high air porosity. Results indicated successful survival of all recipient pigs without jaundice or liver dysfunction. At five weeks post-intervention, the patch was undetectable, with fibroid adhesions forming without duct obstruction. Histological analysis revealed infiltration of inflammatory cells and fibrous tissue. By four months, the patched site resembled the native duct, displaying cuboidal columnar epithelium and positive staining for Cytokeratin CK19, with AST, ALP, and total bilirubin levels remaining within normal ranges. The study highlighted the short-term effectiveness of the BAP patch in promoting duct regeneration without stricture formation. Compared to non-absorbable prosthetic materials prone to complications, the BAP patch cleared from the body within five weeks, leaving no foreign matter.

Another study using a degradable patch was conducted by Tao et al. (2015) [14]. This study developed a porous, biocompatible, and degradable collagen patch that maintained shape in bile for approximately 4 weeks. Collagen, being a major extracellular matrix material, was chosen for its widespread use in tissue engineering, promoting tissue regeneration effectively. The defect reconstruction involved using a collagen patch with a drainage tube and wrapping it with greater omentum to prevent early bile leakage. The study highlighted the challenges of managing biliary strictures and the complications associated with conventional methods like biliary-enteric bypass. Autologous tissue patches and biomaterial-based patches have been proposed, each with its drawbacks concerning degradability and mechanical strength. The collagen membrane, primarily composed of type I and type III collagens, underwent chemical cross-linking and freeze-drying to form suturable membranes. Surgical procedures were conducted accordingly. Results indicated the survival of all pigs post-surgery with no significant adverse effects observed. Histological analysis revealed gradual regeneration of accessory glands and epithelial cells at graft sites within four weeks, demonstrating the collagen patch's effectiveness in inducing regeneration within 12 weeks. The xenogeneic collagen membranes from bovine skin showed no adverse effects and were gradually replaced by regenerated bile ducts. Epithelial cell regeneration occurred more rapidly compared to bioabsorbable polymer patches, highlighting the efficacy of the collagen patch.

Autologous grafts and other prosthetic materials pose drawbacks such as processing time and infection risks, making the BAP patch a promising alternative for emergency cases due to its durability and biocompatibility.

### Prosthetic grafts

The study done by Christensen M., et al. (2005) aimed to explore reconstructing the common bile duct (CBD) using a vascular prosthetic graft, employing a specialized suturing technique and fibrin glue [11]. Utilizing an expanded polytetrafluoroethylene (ePTFE) vascular graft, the investigation sought to assess the feasibility of this approach. Results indicated unaffected liver parameters and leukocytes post-procedures, with no significant weight loss observed. Cholangiography revealed minor intrahepatic dilation. The study demonstrated successful CBD reconstruction without bile leakage into the peritoneal cavity, preserving the sphincter of Oddi. However, its efficacy in preventing reflux into the biliary tree remains uncertain. The method appears promising for reconstructing iatrogenic bile duct injuries or benign strictures. Nevertheless, the vascular graft was consistently found fragile and non-resilient, indicating a need for improved graft materials to maintain elastic properties.

Another study using the prosthetic graft was done by Montalvo-Jave, et al. (2015), which assessed the effectiveness of a polymer-based absorbable bioprosthesis with a bone scaffold [15]. The bioprosthesis, comprising collagen with open pores and a demineralized bone scaffold, coated with epsilon-caprolactone, prevented leakage. Utilizing this bioprosthesis as a scaffold facilitated tissue regeneration without complications at a 6-month follow-up. It has potential applications in replacing bile ducts in various conditions, including cancer and injury. The experimental animals under study did not exhibit any early postoperative complications, including stenosis, bile duct leakage, collection, or death. However, further research is needed to evaluate its efficacy in inflamed or infected conditions, such as bile duct injury post-cholecystectomy. The structure of the prosthesis allows for flexible grafting, enabling connections between multiple ducts in different types of injuries or cancers.

### Bacterial film

Bacterial cellulose, a biological adjunct, has been explored for bile duct repair, offering a promising and cost-effective alternative, similar to its use in urological procedures. Its low toxicity, biocompatibility, and positive results in experimental models support its potential for repairing complex bile duct defects. A study using this material was done by de Abreu GF, et al. (2020). The bioabsorbable cellulose film (BCF) utilized in the research was produced via a biotechnological process from glucose sourced from sugarcane molasses. This material forms a compact film with a thickness of 0.2 mm. In the control group (CG), three deaths occurred within the first week post-surgery, with an additional two fatalities by the third month, attributed to causes such as sepsis from anastomosis stenosis, digestive hemorrhage, and pulmonary embolism [16]. While the BCF effectively prevented bile leakage during common bile duct (CBD) repair due to its dense non-porous structure, this attribute may impede macrophage infiltration and impair fibroblast function, resulting in reduced collagen production and incorporation into the graft material during the healing process. This observation aligns with the prior study, where a similarly dense membrane with estimated low porosity was used for rat abdominal wall repair [34]. Notably, although fibroblast invasion was hindered, the new layers of the intima and adventitia displayed strong adhesion to the luminal surface of the BCF patch during vascular repair in dogs, as reported by previous research [35].

### Heterogeneous materials

Shang H. et al. (2020) used another approach for repairing bile duct injuries using a heterogeneous material. The study utilized an animal-derived artificial bile duct (ada-BD) due to its close resemblance to the bovine ureter and bile duct (BD) in terms of pathological structure, sterile environment, physiological pressure, diameter, thickness, and compliance [17]. Immunological rejection is a major concern in graft transplantation, necessitating the resolution of graft immunogenicity [36,37]. To address this, the immunogenicity of the bovine ureter was effectively removed through the Triton-X-100/phosphate-buffered saline decellularization technique. Subsequent reinforcement through crosslinking with a glutaraldehyde solution enhanced mechanical properties, thermal stability, and anti-calcification features. This resulted in the fabrication of an animal-derived BD scaffold for the study.

All the experimental animals had normal liver function test results, except for the pig in the group with a deficiency in the distal common bile duct, which experienced bile leakage. However, only the levels of ALT on postoperative day 7 showed a substantial increase. The observed elevation in ALT levels on the seventh day was related to hepatic impairment arising from the leakage of bile. Macroscopic and microscopic assessments of the neo-extrahepatic BD revealed no significant differences from a normal duct after 12 months. These findings suggest that the ada-BD, being nonimmunogenic, provides favorable conditions for regenerating autologous BD structures. Consequently, the ada-BD successfully repaired BD segmental defects, achieving outcomes comparable to autologous BD end-to-end anastomosis.

Based on the findings of this review, several graft techniques show significant promise for bile duct repair. Decellularized grafts, such as ureteral and small intestinal submucosa-derived scaffolds, exhibit strong biocompatibility and regenerative potential but require supportive structures like stents to prevent complications. Similarly, bacterial cellulose films demonstrate excellent biocompatibility and healing outcomes, although further optimization is needed to improve integration and reduce the risk of fibrosis. Biodegradable stents combined with autologous tissue also represent a promising approach by eliminating the need for removal and minimizing long-term complications. While no single technique can currently be identified as superior, these methods provide a foundation for future research aimed at translating experimental findings into clinically viable solutions.

### Limitation of study

The primary limitation of this systematic review lies in the variability of methodologies and reporting among the included studies. While the SYRCLE tool provided insights into the risk of bias, the inconsistent quality of reporting, particularly in blinding, allocation concealment, and outcome assessment, limits the reliability of the findings. Furthermore, although qualitative techniques were employed in the data analysis to identify patterns and themes, a comprehensive qualitative synthesis or meta-analysis was not feasible due to the heterogeneity of study designs, interventions, and outcome measures. This prevented a robust comparison of the effectiveness of different bile duct repair techniques. Finally, the limited sample sizes, variability in study duration, and use of different animal models further restrict the generalizability of the conclusions to clinical practice. These limitations underscore the need for standardized methodologies and detailed reporting in future research to allow for more rigorous evaluation.

## Conclusion

In conclusion, the diverse array of techniques explored for bile duct repair offers promising avenues for addressing defects and lesions. From utilizing degradable stents and autologous tissue to creating artificial bile ducts with the body's resources, such as veins and grafts, each method presents unique advantages and considerations. While short-term safety and feasibility have been demonstrated for many of these approaches, there remains a crucial need for long-term observation to ascertain their efficacy beyond one year. Moreover, innovative solutions like decellularized ureteral grafts, autologous neo-bile duct implantation, and collagen membrane patches show considerable potential for clinical application in treating bile duct injuries and stenosis. Additionally, the utilization of novel materials such as bacterial cellulose film highlights the ongoing exploration of biocompatible alternatives. As research continues, these advancements hold promise for improving outcomes and providing effective treatments for patients with bile duct disorders.

## PRISMA 2020 Checklist statement

The authors have read the PRISMA 2020 Checklist, and the manuscript was prepared and revised according to the PRISMA 2020 Checklist.

## Funding sources statement

This research was conducted without any form of direct or indirect financial support.

## CRediT authorship contribution statement

**Anung Noto Nugroho:** Writing – review & editing, Writing – original draft, Visualization, Validation, Supervision, Software, Resources, Project administration, Methodology, Investigation, Funding acquisition, Formal analysis, Data curation, Conceptualization. **Soetrisno Soetrisno:** Project administration, Methodology, Investigation, Funding acquisition, Formal analysis, Data curation, Conceptualization. **Ambar Mudigdo:** Resources, Project administration, Methodology, Investigation, Funding acquisition, Formal analysis, Data curation, Conceptualization. **Kristanto Yuli Yarso:** Writing – review & editing, Writing – original draft, Visualization, Validation, Supervision, Software, Resources, Project administration, Methodology, Investigation, Funding acquisition, Formal analysis, Data curation, Conceptualization. **Dono Indarto:** Resources, Methodology, Investigation, Funding acquisition, Formal analysis, Data curation, Conceptualization. **Akmal Zhahir Wahyudi:** Writing – review & editing, Writing – original draft, Visualization, Validation, Supervision, Software, Resources, Project administration, Methodology, Investigation, Funding acquisition, Formal analysis, Data curation, Conceptualization. **Enrico Ananda Budiono:** Writing – review & editing, Writing – original draft, Visualization, Validation, Supervision, Software, Resources, Project administration, Methodology, Investigation, Funding acquisition, Formal analysis, Data curation, Conceptualization. **Auliya Yudia Yasyfin:** Writing – review & editing, Writing – original draft, Visualization, Validation, Supervision, Software, Resources, Project administration, Methodology, Investigation, Funding acquisition, Formal analysis, Data curation, Conceptualization.

## Ethical approval statement

This study received ethical approval from the Health Research Ethics Committee at Dr. Moewardi General Hospital.

## Declaration of Generative AI and AI-assisted technologies in the writing process

During the preparation of this work, the authors used ChatGPT to assist in the writing process to improve readability and language. After using this tool/service, the authors reviewed and edited the content as needed and took full responsibility for the content of the publication.

## Declaration of competing interest

We declare that there are no affiliations with or involvement in any organization or entity with a financial interest in the subject matter discussed in this manuscript.

## References

[1] Barbier L., Souche R., Slim K., Ah-Soune P. (2014 Sep). Long-term consequences of bile duct injury after cholecystectomy. J Visc Surg [Internet].

[2] Lau W.Y., Lai E.C.H., Lau S.H.Y. (2010 Jan 4). Management of bile duct injury after laparoscopic cholecystectomy: a review. ANZ J Surg [Internet].

[3] Way L.W., Stewart L., Gantert W., Liu K., Lee C.M., Whang K. (2003 Apr). Causes and prevention of laparoscopic bile duct injuries. Ann Surg [Internet].

[4] de’Angelis N., Catena F., Memeo R., Coccolini F., Martínez-Pérez A., Romeo O.M. (2021 Dec 10). 2020 WSES guidelines for the detection and management of bile duct injury during cholecystectomy. World J Emerg Surg [Internet].

[5] Hooijmans C.R., Rovers M.M., de Vries R.B., Leenaars M., Ritskes-Hoitinga M., Langendam M.W. (2014 Dec 26). SYRCLE’s risk of bias tool for animal studies. BMC Med Res Methodol [Internet].

[6] Page M.J., McKenzie J.E., Bossuyt P.M., Boutron I., Hoffmann T.C., Mulrow C.D. (2021 Mar 29). The PRISMA 2020 statement: an updated guideline for reporting systematic reviews. BMJ [Internet].

[7] Liang Y.L., Yu Y.C., Liu K., Wang W.J., Ying J.B., Wang Y.F. (2012). Repair of bile duct defect with degradable stent and autologous tissue in a porcine model. World J Gastroenterol.

[8] Palmes D., Wolters H., Spiegel H.U., M¨ller E., Minin E., Heistermann H.P. (2009 Dec 22). Morphological changes during creation of a neo-bile duct using a vein and a biodegradable endoluminal stent. J Investig Surg [Internet].

[9] Cheng Y., Xiong X.Z., Zhou R.X., Deng Y.L., Jin Y.W., Lu J. (2016). Repair of a common bile duct defect with a decellularized ureteral graft. World J Gastroenterol [Internet]..

[10] Struecker B., Hillebrandt K.H., Raschzok N., Jöhrens K., Butter A., Tang P. (2016). Implantation of a tissue-engineered neo-bile duct in domestic pigs. Eur Surg Res [Internet].

[11] Christensen M., Laursen H.B., Rokkjær M., Jensen P.F., Yasuda Y., Mortensen F.V. (2005 Jun 27). Reconstruction of the common bile duct by a vascular prosthetic graft: an experimental study in pigs. J Hepatobiliary Pancreat Surg [Internet].

[12] Heistermann H.P., Palmes D., Stratmann U., Hohlbach G., Hierlemann H., Langer M. (2006 Jan 9). A new technique for reconstruction of the common bile duct by an autologous vein graft and a biodegradable endoluminal stent. J Investig Surg [Internet].

[13] Aikawa M., Miyazawa M., Okamoto K., Toshimitsu Y., Torii T., Okada K. (2010 Apr). A novel treatment for bile duct injury with a tissue-engineered bioabsorbable polymer patch. Surgery [Internet].

[14] Tao L., Li Q., Ren H., Chen B., Hou X., Mou L. (2015 Apr). Repair of extrahepatic bile duct defect using a collagen patch in a swine model. Artif Organs [Internet].

[15] Montalvo-Javé E.E., Mendoza Barrera G.E., Valderrama Treviño A.I., Piña Barba M.C., Montalvo-Arenas C., Rojas Mendoza F. (2015 Aug). Absorbable bioprosthesis for the treatment of bile duct injury in an experimental model. Int J Surg [Internet].

[16] de Abreu G.F., Batista L.L., Adeodato D.C., de Albuquerque A.V., Ferraz-Carvalho R.S., de Lima R.P. (2020 Sep 5). Use of bacterial cellulose film for repair of bile duct injury in pigs. J Biomater Appl [Internet].

[17] Shang H., Zeng J.P., Wang S.Y., Xiao Y., Yang J.H., Yu S.Q. (2020 Dec 14). Extrahepatic bile duct reconstruction in pigs with heterogenous animal-derived artificial bile ducts: a preliminary experience. World J Gastroenterol [Internet].

[18] Pérez Alonso A.J., Del Olmo Rivas C., Romero I.M., Cañizares Garcia F.J., Poyatos P.T. (2013 Jan). Tissue-engineering repair of extrahepatic bile ducts. J Surg Res [Internet].

[19] Li Q., Tao L., Chen B., Ren H., Hou X., Zhou S. (2012 Jun). Extrahepatic bile duct regeneration in pigs using collagen scaffolds loaded with human collagen-binding bFGF. Biomaterials [Internet].

[20] Nau P., Liu J., Ellison E.C., Hazey J.W., Henn M., Muscarella P. (2011 Aug). Novel reconstruction of the extrahepatic biliary tree with a biosynthetic absorbable graft. HPB [Internet].

[21] Miyazawa M., Torii T., Toshimitsu Y., Okada K., Koyama I., Ikada Y. (2005 Jun). A tissue-engineered artificial bile duct grown to resemble the native bile duct. Am J Transplant [Internet].

[22] Rosen M., Ponsky J., Petras R., Fanning A., Brody F., Duperier F. (2002). Small intestinal submucosa as a bioscaffold for biliary tract regeneration. Surgery.

[23] Mercado M.A., Chan C., Orozco H., Cano-Gutiérrez G., Chaparro J.M., Galindo E. (2002). To stent or not to stent bilioenteric anastomosis after iatrogenic injury: a dilemma not answered?. Arch Surg.

[24] Cushieri A., Baker P.R., Anderson R.J.L., Holley M.P. (1983). Total and subtotal replacement of the common bile duct: effect of transhepatic silicone tube stenting. Gut.

[25] Vakil N., Gross U., Bethge N. (1999). Human tissue responses to metal stents. Gastrointest Endosc Clin N Am.

[26] Lee M.J., Dawson S.L., Mueller P.R., Hahn P.F., Saini S., Lu D.S.K. (1994). Failed metallic biliary stents: causes and management of delayed complications. Clin Radiol.

[27] Tashiro H., Ogawa T., Itamoto T., Ushitora Y., Tanimoto Y., Oshita A. (2009). Synthetic bioabsorbable stent material for duct-to-duct biliary reconstruction. J Surg Res.

[28] De Reuver P.R., Busch O.R.C., Rauws E.A., Lameris J.S., Van Gulik T.M., Gouma D.J. (2007). Long-term results of a primary end-to-end anastomosis in peroperative detected bile duct injury. J Gastrointest Surg.

[29] Badylak S. (2004 Apr). Xenogeneic extracellular matrix as a scaffold for tissue reconstruction. Transpl Immunol [Internet].

[30] Gilbert T., Sellaro T., Badylak S. (2006 Mar 7). Decellularization of tissues and organs. Biomaterials [Internet].

[31] Gómez N.A., Zapatier J.A., Vargas P.E. (2004 Apr). Re: “small intestinal submucosa as a bioscaffold for biliary tract regeneration”. Surgery [Internet].

[32] Wills V.L., Gibson K., Karihaloo C., Jorgensen J.O. (2002 Mar 19). Complications of biliary T-tubes after choledochotomy. ANZ J Surg [Internet]..

[33] Faulk D.M., Wildemann J.D., Badylak S.F. (2015). Decellularization and cell seeding of whole liver biologic scaffolds composed of extracellular matrix. J Clin Exp Hepatol.

[34] Silveira R.K., Coelho A.R.B., Pinto F.C.M., de Albuquerque A.V., de Melo Filho D.A., de Andrade Aguiar J.L. (2016). Bioprosthetic mesh of bacterial cellulose for treatment of abdominal muscle aponeurotic defect in rat model. J Mater Sci Mater Med.

[35] Marques S.R.D.B., Lins E.M., Aguiar J.L.D.A., Albuquerque M.C.S., Rossiter R.D.O., Montenegro L.T. (2007). A new vascular substitute: femoral artery angioplasty in dogs using sugarcane biopolymer membrane patch - hemodynamic and histopathologic evaluation. J Vasc Bras.

[36] Lim E.J., Chin R., Nachbur U., Silke J., Jia Z., Angus P.W. (2015 Sep 21). Effect of immunosuppressive agents on hepatocyte apoptosis post-liver transplantation. Dehghani F, editor. PLoS One [Internet].

[37] Perry I., Neuberger J. (2004 Dec 14). Immunosuppression: towards a logical approach in liver transplantation. Clin Exp Immunol [Internet].

